# Severe toxic rhabdomyolysis under combined palbociclib and simvastatin treatment: A case report

**DOI:** 10.3389/fonc.2022.1026434

**Published:** 2022-12-14

**Authors:** François Poumeaud, Anna Fontanier, Jérémie Dion, Quentin Mathevet, Olivier Cointault, Emmanuelle Uro-Coste, Céline Marty, Florence Dalenc, Pierre Girardie, Anaïs Rataboul

**Affiliations:** ^1^ Department of Medical Oncology, Institut Claudius Regaud - Institut Universitaire du Cancer de Toulouse - Oncopole, Toulouse, France; ^2^ Department of Clinical Pharmacy, Institut Claudius Regaud - Institut Universitaire du Cancer de Toulouse - Oncopole, Toulouse, France; ^3^ Department of Internal Medicine and Clinical Immunology, Centre Hospitalier Universitaire Toulouse, Toulouse, France; ^4^ Department of Nephrology, Centre Hospitalier Universitaire Toulouse, Toulouse, France; ^5^ Department of Anatomo-pathology, Institut Universitaire du Cancer de Toulouse – Oncopole, Toulouse, France; ^6^ Department of Neurology, Centre Hospitalier Universitai Toulouse – Purpan, Toulouse, France

**Keywords:** rhabdomyolysis, palbociclib, simvastatin, interaction, myositis, plasmatic exchange

## Abstract

We report the fourth described case of severe toxic rhabdomyolysis occurring in an 81-year-old woman caused by the concomitant administration of palbociclib taken at the usual dosage (125 mg per day) and simvastatin. To the best of our knowledge, this is the first reported case successfully treated by plasma exchanges, with complete functional recovery within two months. The severity of this case justifies further consideration of pharmacokinetic interactions between palbociclib or other CDK-4-6 inhibitors and statins, which potentially increase the risk of an adverse event.

## 1 Introduction

Cyclin-dependent kinase 4 and 6 (CDK4/6) inhibitors combined with endocrine therapy (aromatase inhibitor or fulvestrant) are the standard-of-care for estrogen receptor (ER)-positive/HER2-negative metastatic breast cancer (MBC) without visceral crisis/failure and result in longer progression-free and overall survival with a good toxicity profile (1, 2). Palbociclib is one of the commonly used oral CDK4/6inhibitors. Inter-individual variations in exposure to the drug are large as it is extensively metabolized by cytochrome P450 3A4 (CYP3A4) and its brain penetration is limited by efflux transporters (3). Specific precautions must therefore be taken to avoid any potential interactions with other substances metabolized by CYP3A4.

Statins are widely prescribed to the general population, especially simvastatin which is metabolized by CYP3A4. Consequently, CYP3A4 inhibitors, such as palbociclib, may increase the plasma concentration of simvastatin, thus increasing the risk of an adverse reaction. To date, four cases of severe toxic rhabdomyolysis related to inhibitors of cyclin-dependent kinase 4 and 6 drugs have been reported in the literature (**Table 1**). All of these occurred with the association of statin-drugs (3 with simvastatin, 1 with atorvastatin) with conventional doses of palbociclib (3 cases) (4–6) or ribociclib (1 case) (7). It seems unlikely thatCDK4/6 inhibitors would cause inflammatory myopathy or rhabdomyolysis since no such cases have been reported for either palbociclib (8–10), ribociclib (11, 12) or abemaciclib. Here, we describe a case of severe toxic rhabdomyolysis due to the concomitant exposure to palbociclib and simvastatin in a woman suffering from MBC, and the first report of its successful treatment *via* a plasma exchange-based therapy.

**Table 1:** Reported toxic myositis in patients treated with cyclin-dependent inhibitors.

Cancer	Patient characteristic	CDK4-6 inhibitor	Statin	Severity of rhabdomyolysis NCI-CTC V4	Reference
Advanced non-small cell lung cancer		palbociclib (125 mg/day, 21 days/28days)	simvastatin (80 mg/day)	Grade 4	(4)
ER+ HER2-, metastatic breast cancer	Woman (60)		atorvastatin (40 mg/day)	Grade 5	(5)
	Woman (71)		simvastatin (40 mg/day)	Grade 4	(6)
	Woman (68)	ribociclib (600 mg/day, 21 days/28days)	simvastatin (40 mg/day)	Grade 4	(7)

## 2 Case description

An 81-year-old woman presented with a six-day history of progressive muscle pain and progressive weakness of the lower limbs. She had taken simvastatin for over twenty years, and simvastatin 20 mg plus ezetimibe 10 mg for five years to treat dyslipidemia. She had also been prescribed apixaban, venlafaxine, L-thyroxine and allopurinol for several years. She presented with an ER+/HER2-negative *de novo* MBC with bone extension in the summer of 2021. She had a slightly increased tumor marker (CA 15.3 241 kU/L) at baseline.

A first-line metastatic combination treatment with an aromatase-inhibitor (letrozole 2,5 mg per day) and CDK4-6 inhibitor (palbociclib125 mg per day, 21 days/28) was initiated. After 20 days of concomitant treatment, she presented with a generalized mild-intensity myalgia associated with the onset of a proximal motor deficit of the lower limbs, which subsequently extended to the upper limbs and developed into discrete myalgia. At day 23 after treatment initiation, she presented with proximal muscle weakness affecting both the lower and upper limbs, she was unable to brush her hair or to straighten-up. She then had a fall and laid on the floor for one hour before being transferred to the nearby emergency unit.

In the emergency unit, she was diagnosed with proximal tetraparesis and intense rhabdomyolysis (CPK 18.000 UI/L, approximately 90 times over the upper limit of 170 UI/L) which was inconsistent with the absence of extensive hematoma. There was no alteration of glomerular filtration.

Timeline: The patient initiated palbociclib on December 4^th^ for 21 days. At day 23, she exhibited first muscle weakness and was admitted to the emergency unit. Corticotherapy and saline hydration were initiated at day 28 and plasmatic exchanges at day 32 for three sessions.

## 3 Diagnostic assessment

When transferred to the medical oncology department, the patient presented with a severe proximal tetraparesis predominantly involving the proximal inferior-muscle girdle, which was associated with a dropped head syndrome secondary to severe weakness of the neck extensors but not affecting the diaphragm or deglutition. Cranial nerve examination and sensory evaluation were non-pathological. A careful clinical examination found no evidence of cutaneous lesions consistent with dermato-polymyositis.

Three hypotheses were explored to substantiate the etiology of the myogenic deficit with concomitant rhabdomyolysis:

-In light of the active MBC, we initially suspected an auto-immune disorder and paraneoplastic syndrome. However, her imaging data pointed to a controlled disease, no visceral lesions, no new bone lesions and a biologically stable disease (CA 15.3 level stable at 249 kU/L despite altered hepatic function). Notably, thorax tomodensitometry revealed no pulmonary lesions compatible with diffuse interstitial lung disease. An extensive screen for a panel of myositis autoantibodies was negative (see **Supplementary Data**). Free carnitine was dramatically increased and significant acylcarnitinemia was compatible with massive rhabdomyolysis. These findings were consistent with a diagnosis of myositis.

A bacterial screen with several pairs of negative hemocultures to test for a suspected infectious myositis was negative. Tests for viral primo-infections or reactivation were tested, and were negative (see **Supplementary data**). Viral PCR tests were all negative and/or below the significance threshold and were therefore deemed to be clinically irrelevant. Furthermore, there was no evidence that the patient had contracted a COVID-19 infection during her hospitalization.

We finally suspected a toxic rhabdomyolysis due to a palbociclib/simvastatin interaction, which appeared to be consistent with the timing of the symptoms reported by the patient. Further discussion with the patient revealed she was self-medicating with fenugreek (trigonellafoenum-graecum), an orexigenic phytotherapy with cytochrome inhibitory properties, which may have promoted the interaction between the CDK4-6 inhibitor and the statin.

**Figure 1:**
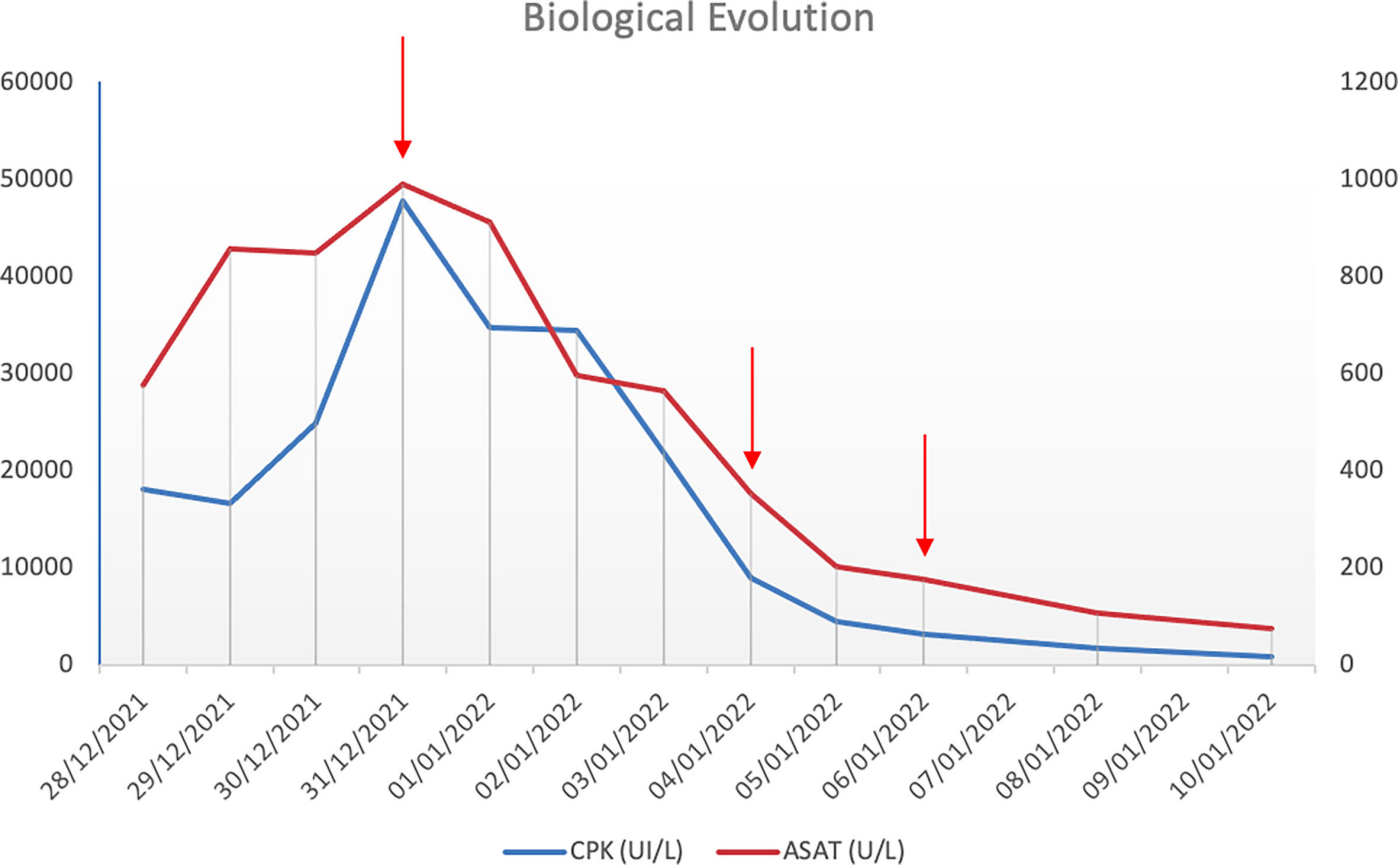
Evolution of CPK and ASAT after three plasma exchange cycles (red arrows). The natural evolution of CPK and ASAT levels before initiation of plasmatic exchanges is symbolized by red arrows. As illustrated, three plasmatic exchanges led to a significant decrease in both CPK and ASAT levels.

Despite the administration of intravenous corticotherapy (1.5 mg/kg/day) as well as a appropriate hydration with 2L/day of sodium chloride, her CPK levels continued to increase to 47.000 UI/L, presumably because palbociclib and simvastatin bind strongly to plasma proteins (85 and 95%, respectively). We therefore performed a series of plasma exchanges to wash out any residual bound palbociclib and simvastatin. The patient underwent three sessions of apheresis on alternative days (see **Figure 1**). We performed a selective plasma separation with a pore size that was small enough for the transfer of albumin but not of higher molecular weight molecules. Two plasma volumes were treated during each session. Tolerance was excellent with no adverse event occurring during or after hospitalization. One month after discharge, the patient was able to walk unassisted in her home, to groom herself and had no muscular impediments. At two months from discharge, she presented no sequelae and was perfectly autonomous in her daily life activities. Palbociclib was definitively discontinued, and the patient was treated with letrozole alone. CT-scan at three months showed progression of the bone metastasis which was treated with tamoxifen, followed by capecitabine for seven months due to disease progression. This patient is currently living independently at home and can perform her daily activities without material assistance.

**Figure 2:**
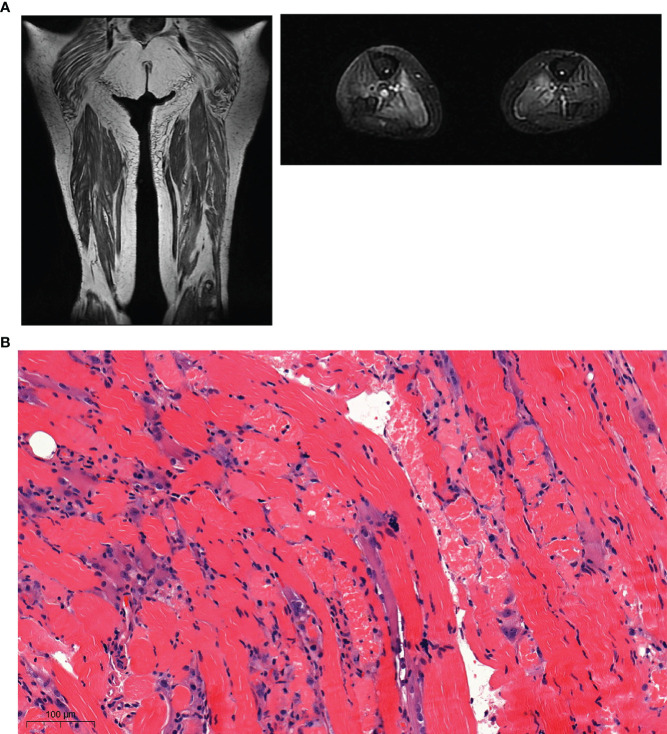
**(A)** Magnetic Resonance Imaging (MRI) of muscles of lower limbs, T1 Coronal and T2 axial reconstruction. MRI shows pathological bilateralhypersignal of proximal muscles: gluteal muscles, medial gastrocnemius muscles, solar muscles with relative respect of semitendinosus muscles. **(B)** Immuno-histo-chemical staining of the muscle biopsy. Standard muscle biopsy stain exhibiting muscle atrophy, degeneration, necrosis, macrophagic infiltration and muscle fiber irregularity.

Given the long waiting list to obtain an MRI test, the diagnosis was confirmed retrospectively by MRI of the lower-limb muscles at the end of plasmatic exchanges. It showed bilateral and symmetrical hyper-intense signals on short-tau-inversion-recovery (STIR) sequences, on most of the muscles explored. This was compatible with muscular oedema, specifically involving the medial gastrocnemii and solaris muscles (see **Figure 2A**). An MRI-guided muscle biopsy was therefore performed on the left quadricipital muscle. This revealed a muscle inflammation with macrophage infiltration, fibrillary involution and necrosis of myocytes, thereby confirming the diagnosis of necrosing myositis (see **Figure 2B**).

## 4 Discussion

Statins such as simvastatin and atorvastatin are cleared *via* a CYP3A4-mediated hepatic metabolic pathway. Since CDK 4-6 inhibitors such as palbociclib and ribociclib are time-dependent CP3A4-inhibitors, they may decrease hepatic clearance and increase the plasma concentrations of statins, thereby potentially exposing patients to toxicities (13). *In silico* pharmaco-kinetic modeling of atorvastatin and palbociclib found a CYP3A4-mediated increase in atorvastatin-lactones (associated with statin-induced-myopathy and rhabdomyolysis) when atorvastatin was co-administered with palbociclib (14). Since simvastatin and atorvastatin share the same CYP3A4 metabolic pathway, these results obtained *in silico* may also extrapolate to simvastatin.

In accordance with procedural recommendations, we reported the case to the pharmacovigilance department who further declared it to the French National Agency for Medicines and Health products Safety (ANSM). In France, no other cases of rhabdomyolysis have been reported with palbociclib or when palbociclib is combined with simvastatin or more generally when CK4/6 inhibitors and statins are given concomitantly. The pharmacovigilance department of our hospital has estimated that the area under curve (AUC) of simvastatin increased to 1.6 when palbociclib was added. The intrinsic causality of rhabdomyolysis by the combination of palbociclib and simvastatin was therefore considered very plausible (I5 score) (15). Using the Adverse Drug Reaction Probability Scale evaluates the interaction as plausible, with a score of 5 (16). As pitavastatin and fenofibrate are not CYP3A4 substrates their use should be recommended, instead of simvastatin or atorvastatin, when considering a combination treatment withCDK4-6 inhibitors (13).

*In vitro*, letrozole is an inhibitor of CYP450 2A6 and 2C19 but its clinical relevance is still unknown. Moreover, even if letrozole is a CYP3A4 substrate, and although an inhibitor such as palbociclib may decrease its metabolism and increase blood exposure, cases of myalgia have been described but no rhabdomyolysis. Finally, the treatment was not discontinued during hospitalization. Therefore, we cannot consider letrozole as the suspect cause of the adverse effect itself or of its onset.

In the present case, the co-treatment with fenugreek (taken for only two days) did not likely contribute to worsening the interaction, since several publications have reported that its *in vivo* metabolic inhibition mediated by CYP3A4 is not substantial (17–19). However, fenugreek contains quercetin, which is a strong CYP3A4 inhibitor and has been shown to increase the AUC of cyclosporin (20). In our patient, fenugreek may have decreased the metabolism of palbociclib and the simvastatin and therefore contributed to worsening the undesirable effect. Caution should thus be exercised when fenugreek is co-administered with CYP34A substrates.

Data concerning the increased risk of rhabdomyolysis with co-administration of statin and ezetimibe are conflicting. Whilst one case study reported on rhabdomyolysis occurring with the combination of statin and ezetimibe (21), a larger review of the literature found no increased risk of this drug combination (22, 23). The current recommendation to initiate ezetimibe for statin-induced myopathy (24), would perhaps also requires further investigation. Whether rhabdomyolysis may be attributed to ezetimibe in our patient cannot be chronologically evaluated, since it was introduced concomitantly with simvastatin. Moreover, pharmacologically ezetimibe is an inhibitor of intestinal cholesterol transport and has no physiological role in muscles. Finally, the literature does not support its involvement given the few cases reported when administered as a monotherapy. The statin would therefore seem to be incriminated in our case.

As in the other case reports, our patient received steroids (1.5 mg/kg/day intravenously) and abundant hydration with sodium chloride (2L per day) to prevent kidney failure. A Danish team administered experimental intravenous immunoglobulins (IgIV) for five days, while waiting for the auto-immune laboratory results (6). In the absence of clear supportive paraneoplastic and auto-immune evidence, and in light of the considerable risk of acute renal failure due to the active rhabdomyolysis (25) as well as the significant proteinuria that developed during hospitalization, we did not opt forIgIV. To our knowledge, however, we are the first team to report on the successful recovery of a patient by an experimental plasma exchange protocol, based on the pharmacokinetic properties of the drugs suspected of inducing the adverse effect.

## 5 Conclusion

This is the fourth reported case of severe toxic rhabdomyolysis occurring under palbociclib and statins (**Table 1**). All of them occurred with the co-administration of statin-drugs (3 with simvastatin, 1 with atorvastatin) and a conventional dosage of palbociclib (3 cases) (4–6) or ribociclib (1 case) (7). No such cases have to date been reported with abemaciclib. We also report on what we believe is the first successful use of plasma exchange for this indication. The current French RCP and FDA prescribing information for palbociclib mentions changes in plasma concentrations for CYP3A4 substrates with a narrow therapeutic index. Statins are not among these drugs and are therefore not mentioned. Substrates with strong adverse effects should be considered in the same way as those mentioned in the prescribing information and require close therapeutic monitoring and dose adjustment. Further clarification of this issue now required.

## Data availability statement

The original contributions presented in the study are included in the article/**Supplementary material**. Further inquiries can be directed to the corresponding author.

## Ethics statement

Written informed consent was obtained from the individual(s) for the publication of any potentially identifiable images or data included in this article.

## Author contributions

FP diagnosed and treated the patient, wrote, edited and corrected the main part of the article. AF wrote and corrected some parts of the manuscript. JD helped to diagnose the patient and corrected the manuscript. QM corrected some parts of the manuscript. OC performed the plasma exchanges and corrected some part of the manuscript. EU-C performed the analysis of the biopsy and corrected some parts of the manuscript. CM wrote and corrected some parts of the manuscript. FD wrote and corrected some parts of the manuscript. PG helped to diagnose the patient, wrote and corrected some parts of the manuscript. AR wrote and corrected some parts of the manuscript and handled the correspondence. All authors contributed to the article and approved the submitted version.
