# Corrigendum: Severe toxic rhabdomyolysis under combined palbociclib and simvastatin treatment: A case report

**DOI:** 10.3389/fonc.2023.1149399

**Published:** 2023-02-07

**Authors:** François Poumeaud, Anna Fontanier, Jérémie Dion, Quentin Mathevet, Olivier Cointault, Emmanuelle Uro-Coste, Céline Marty, Florence Dalenc, Pierre Girardie, Anaïs Rataboul

**Affiliations:** ^1^ Department of Medical Oncology, Institut Claudius Regaud - Institut Universitaire du Cancer de Toulouse - Oncopole, Toulouse, France; ^2^ Department of Clinical Pharmacy, Institut Claudius Regaud - Institut Universitaire du Cancer de Toulouse - Oncopole, Toulouse, France; ^3^ Department of Internal Medicine and Clinical Immunology, Centre Hospitalier Universitaire Toulouse, Toulouse, France; ^4^ Department of Nephrology, Centre Hospitalier Universitaire Toulouse, Toulouse, France; ^5^ Department of Anatomo-pathology, Institut Universitaire du Cancer de Toulouse –Oncopole, Toulouse, France; ^6^ Department of Neurology, Centre Hospitalier Universitai Toulouse –Purpan, Toulouse, France

**Keywords:** rhabdomyolysis, palbociclib, simvastatin, interaction, myositis, plasmatic exchange

**Error in Author List**

In the published article, there was an error in the author list, and author’s first and last names were erroneously inverted. The corrected author list appears below.

François Poumeaud, Anna Fontanier, Jérémie Dion, Quentin Mathevet, Olivier Cointault, Emmanuelle Uro-Coste, Céline Marty, Florence Dalenc, Pierre Girardie6 and Anaïs Rataboul1*

The authors apologize for this error and state that this does not change the scientific conclusions of the article in any way. The original article has been updated.

