# Supplementary material for: Severe toxic rhabdomyolysis under combined palbociclib and simvastatin treatment: A case report
Source: Front Oncol. 2022 Dec 14;12:1026434. doi: 10.3389/fonc.2022.1026434 (PMC9795206; doi:10.3389/fonc.2022.1026434)
Supplement: Supplementary file 1 [file DataSheet_1.docx]

**Supplementary Data**

Autoimmunity screening performed: anti-SSA, anti-SSB, anti-Sm, anti-SmRNP, anti-RNP, anti-Scl70, anti-Jo1, anti-centromeres, anti-ribosome, anti-double strand DNA, anti-synthetase dot, anti-Jo1 dot, anti-PL7 dot, anti-PL12 dot, anti-synthetase EJ, anti-mi2, anti-MDA5, anti-TIF1 gamma, anti-SAE1 dot, anti-SAE2, anti-NXP2, anti-SRP, anti-hMGCo reductase, anti-TRIM21 (SSA-52), anti-ANCA, anti-mitochondria, anti-smooth muscles, anti-LKM, anti-cytosol dot.

Bacterial screening: CMV, EBV, HSV, VZV, Parvovirus B19, viral hepatitis (VHA, VHE, VHB, VHC) and HIV.
